# Frailty and diabetes in older adults: Overview of current controversies and challenges in clinical practice

**DOI:** 10.3389/fcdhc.2022.895313

**Published:** 2022-08-19

**Authors:** Mohd Zaquan Arif Abd.Ghafar, Mark O’Donovan, Duygu Sezgin, Elizabeth Moloney, Ángel Rodríguez-Laso, Aaron Liew, Rónán O’Caoimh

**Affiliations:** ^1^ Faculty of Medicine, Universiti Teknologi MARA (Sungai Buloh), Selangor, Malaysia; ^2^ Geriatrics Unit, Selayang Hospital, Selangor, Malaysia; ^3^ Department of Geriatric Medicine, Mercy University Hospital, Cork, Ireland; ^4^ Health Research Board Clinical Research Facility, University College Cork, Cork, Ireland; ^5^ School of Nursing and Midwifery, Aras Moyola, National University of Ireland Galway, Galway, Ireland; ^6^ CIBERFES (Área temática de Fragilidad y Envejecimiento Saludable del Centros de Investigación Biomédica en Red), Instituto de Salud Carlos III, Madrid, Spain; ^7^ Department of Endocrinology, National University of Ireland Galway, Galway, Ireland

**Keywords:** frailty, diabetes mellitus, pre-frailty, geriatrics, endocrinology, comprehensive geriatric assessment, ageing, overview

## Introduction

This article examines the challenges healthcare professionals face when addressing the modern and often twin epidemics of frailty and diabetes mellitus (DM) in ageing societies. Frailty is a complex, multisystem, age-associated syndrome that increases vulnerability to functional decline and adverse events, including death (1, 2). DM is a metabolic disease of defective insulin secretion in response to glucose and impaired insulin sensitivity defined by hyperglycaemia and, similar to frailty, results from the complex interplay of genetic and acquired factors (3).

Both conditions are highly prevalent with age. Global frailty prevalence is estimated at between 12-24% in older (age ≥50 years) community-dwellers (4), though proportions vary widely depending on the sampling frame, participant characteristics and definitions used (5). DM is an epidemic disease with a worldwide prevalence of 20-25% among those ≥70 years (6). Like frailty, DM is associated with increased disability and mortality in older people (7). In developed countries, more than half of the population with DM are aged ≥65 years (8), and it is estimated that the prevalence of frailty is 3-5 fold higher among people with DM than those without (9, 10). Alone and in combination, DM and frailty significantly impact health service provision (11), increasing total healthcare costs (12–14). Frailty and DM together in combination, negatively impact mortality, psychosocial wellbeing and quality of life (15, 16).

The objectives of this article are to provide an up-to-date, evidence-based overview of the relationship between DM and frailty in older adults, specifically examining knowledge gaps and the unique challenges when these conditions co-exist and what clinicians and healthcare systems can do better to address them.

## Understanding the relationship between frailty and DM

The relationship between frailty and DM is complex and several questions remain unanswered, particularly as to whether it is truly a bi-directional association (17, 18). There is however, strong evidence that DM is implicated in the development of frailty. Hyperglycaemia is linked with incident frailty, and glycated haemoglobin levels (HbA1c) are associated with frailty severity (19). Vascular complications from type 2 DM are also associated with inactivity and physical and cognitive decline, suggesting that frailty and its related syndromes are directly implicated in to the end-organ damage from DM (20–23). It is no surprise then that DM is associated with an increased risk of frailty (24) or that longitudinal studies suggest that DM is a predictor of transitioning from lower to higher frailty levels (25–29). Questions remain however, as to whether approaches to improve glycaemic control can reduce incident frailty and transitions over time or indeed reverse early stage (mild) frailty (18).

Similarly, it is unclear if frailty leads to DM or is just associated with worse outcomes in those with established DM. For example, while frailty is recognised as an independent risk factor for morbidity and mortality in people with DM (30), it is not yet known whether frailty is a determinant of HbA1c levels or if the management of frailty influences DM care (18). Further, whether frailty can directly lead to the development of DM is unknown, though recent studies suggest that frailty is associated with incident type 2 DM in older community-dwellers (aged ≥65) (31), likely related to the effect of frailty on ageing muscle (32).

Indeed, frailty, DM and sarcopenia (which is closely associated with frailty) share common physiological mechanisms and pathological changes (33). In older people, loss of muscle mass is accompanied by a relative increase in visceral fat, described as ‘sarcopenic obesity.’ Insulin resistance is hypothesized to result from sarcopenic obesity and mitochondrial dysfunction (34). Low levels of testosterone and insulin-like growth factor are associated with insulin resistance and type 2 DM, and with decreased protein synthesis and muscle mass, which characterizes sarcopenia and the development of physical frailty (35, 36). Beyond sarcopenia, other potential shared mechanisms include inflammation and vitamin D deficiency. A chronic state of low-grade inflammation and oxidative stress are associated with both the evolution of DM (37–39) and frailty (40). Vitamin D deficiency is associated with frailty and subsequent falls and functional decline (41, 42), but also B-cell dysfunction, insulin resistance and inflammation that may result in type 2 DM (43, 44). Further research, both basic science and clinical studies are required to better understand the significance of shared pathophysiological risk factors and features, and whether the relationship is truly bi-directional.

**Figure 1:**
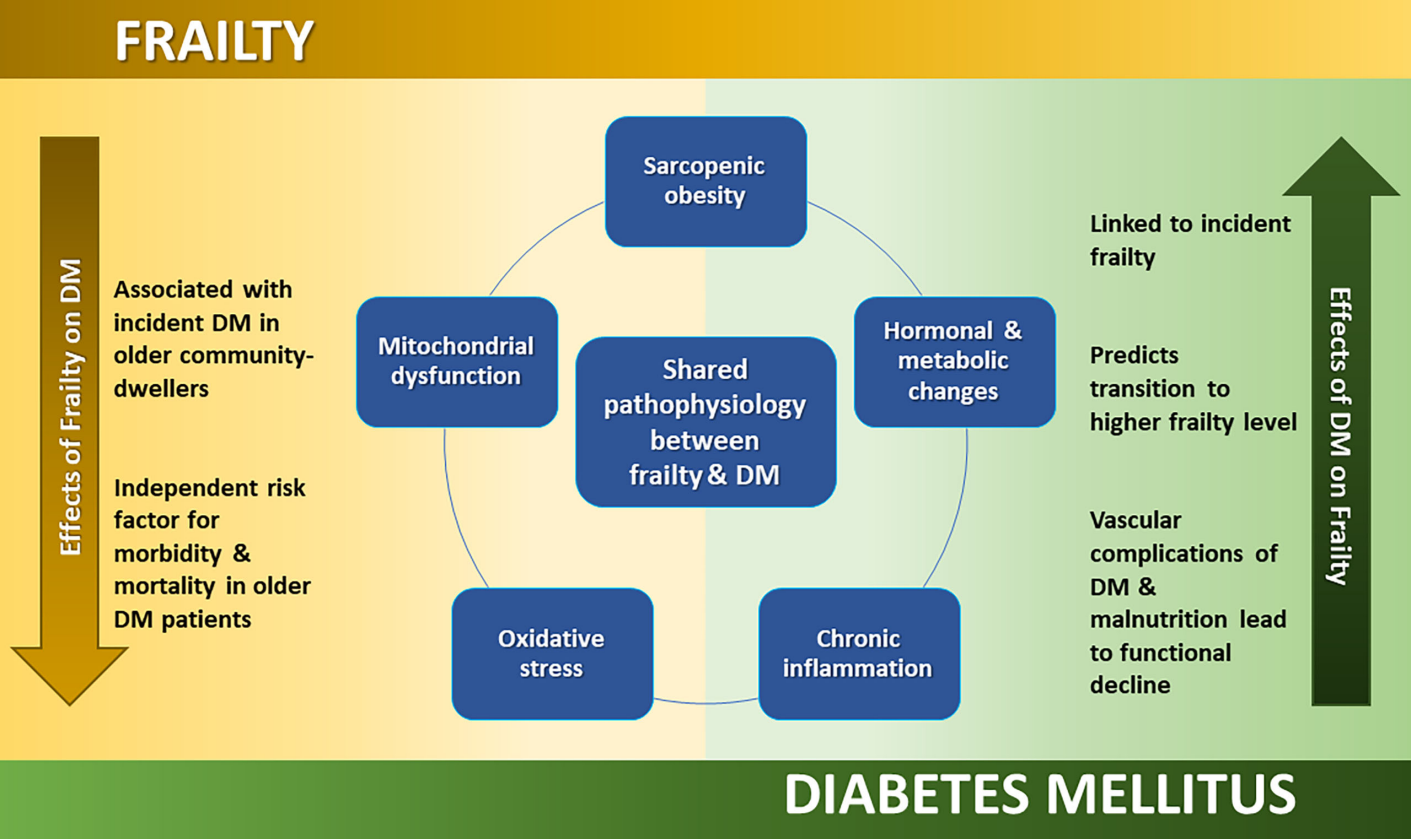
Diagram 1. Overview of shared pathophysiology and differential outcomes of DM and frailty.

A brief overview of the shared pathophysiology and differential outcomes of DM and frailty are presented in **Figure 1**.

## Screening, assessment and management of frailty in diabetes

Detecting frailty in older people with DM is important to target interventions that reduce functional decline and risk of disability (45). These are identified by assessing key factors associated with frailty including physical performance, mental health, cognition and nutrition. Early identification of frailty i.e., pre-frailty, a potentially reversible condition (46, 47), is preferable. Brief, feasible and validated tools are recommended for opportunistic screening or case-finding, followed by confirmation with multidimensional assessment (48, 49). A variety of frailty assessment tools have been examined in people with DM. A recent systematic review found that the Fried Phenotype, describing physical frailty (50), is the most commonly used to approach classify frailty in people with DM (51). Multiple (n=20) measures were identified, though there was marked heterogeneity between studies (51), suggesting the need for more research to identify the psychometric properties of an optimal instrument to measure frailty in DM in different settings. Similarly, few papers have reported the clinimetric performance of different frailty assessment tools in people with DM (19), highlighting the requirement for diagnostic accuracy studies, specifically examining frailty in DM and raising the potential need for bespoke screening instruments.

Comprehensive Geriatric Assessment (CGA) is central to confirming frailty in older patients and tailoring care for those with DM and frailty. CGA is a multidimensional evaluation across interrelated domains (including physical, cognitive, psychological, functional and social), conducted by a team of specialist healthcare professionals (doctors, nurses, therapists, social workers and others). Decision-making is shared with patients and/or caregivers to improve quality of life and functional status. The goal of CGA in frail patients with DM is to ensure risk factors and possible complications are addressed, improve glycaemic control, avoid or minimize hypoglycaemic episodes, and preserve the capability of patients to self-manage (52, 53).

Management of frailty in people with DM should be directed by CGA. However, relatively few studies have examined specific approaches to address frailty in this population, instead focusing on how best to control diabetes. There is evidence that optimal nutrition, exercise and possibly some pharmacological therapies (such as testosterone) may be beneficial in pre-frail or frail older adults (54–57), although these are not specific to people with DM, suggesting the need for bespoke research. Such studies are planned including the EXPLODE randomized controlled trial examining the feasibility and acceptability of gym-based resistance exercise to prevent frailty in older people with DM (58).

## Management of diabetes in frail older people

### General principles

The management of DM in those with established frailty is challenging (8) as clinical presentation, healthcare costs, psychosocial factors, resource availability and treatment options vary markedly in this group (15, 16, 59). Treatment should be holistic and individualized using both pharmacological and non-pharmacological approaches, balancing the benefits of intensive glycaemic control versus the risks of hypoglycaemia. Issues identified in a CGA including polypharmacy, existing comorbidities, cognitive and functional status, and the availability of social supports should be considered (60–62). Step-wise frameworks to help individualise glycaemic treatment in partnership with patients are also required (61). Current guidelines recommend less stringent glycaemic control targets in frail older adults with DM, as strict control is associated with hypoglycaemia and functional decline. Hypoglycaemia in particular, is associated with an increased risk of mortality, cognitive decline, falls and fractures, and indeed the development of frailty (63–65). However, hyperglycaemia, leading to acute complications such as dehydration, poor wound healing and hyperglycaemic hyperosmolar coma, should likewise be avoided (60, 62). A recent expert consensus statement on the management of older people with type 2 DM suggests that HbA1c should be targeted as follows: HbA1c <58mmol/mol in those non-frail, pre-frail or mildly frail adults, to 59-64mmol/mol for moderate frailty and 64-69mmol/mol in those with severe frailty and reduced life expectancy (69). However, as yet these serve only as guidance with little evidence that adherence to these targets improves outcomes. Further, there is a need to understand if there is U-shaped relationship between HbA1c levels and outcomes such as mortality in patients with DM and frailty (51).

## Non-pharmacological: nutrition and exercise

Given the proposed bi-directional relationship between sarcopenia and DM (66) and the high prevalence of sarcopenia among older adults with DM (67), these patients should have individualised nutritional therapy, taking into account their physical and cognitive status, food preferences, economic and social situation, religion and culture (68). While obese older adults with DM may benefit from modest dietary restrictions, this may not be in the best interest of all frail older adults. For those with more advanced frailty, which is itself characterized by malnutrition, sarcopenia and weight loss, the focus should be on maintaining weight (69). Hence, some experts suggest a shift in focus towards optimal nutrition with adequate calorie and protein intake to prevent and treat frailty and sarcopenia in DM (70). Exercise interventions consisting of resistance and balance training are associated with fewer falls, improved functional ability and reduced risk of immobility in physically frail older adults (54, 55). In older adults with frailty and DM, physical interventions should be tailored to individuals’ physical status (71). The International Diabetes Federation (IDF) recommends referral to community-supervised walking schemes and community-based group exercise and fitness programs where available (68).

## Pharmacological therapy

While overtreatment is not uncommon, consensus statements and guidelines still recommend tight glycaemic control using available treatments in fit/non-frail older adults. In contrast, deintensification or simplification of complex regimens is recommended for older adults with multiple complications and reduced function or those at end of life (60, 69, 72). When starting pharmacological treatment, the principle of ‘start low, go slow’ applies (53). Metformin remains the first-line antihyperglycaemic agent in older adults with type 2 DM, if tolerated, following frailty status-tailored lifestyle management. It is contraindicated in advanced renal insufficiency and must be used with caution in older patients with congestive heart failure and hepatic impairment (60, 62, 68). Oral antihyperglycaemic agents with lower hypoglycaemic risks such as glucagon-like peptide-1 (GLP-1) receptor agonists, sodium-glucose co-transporter-2 (SGLT-2) inhibitors and dipeptidyl peptidase-4 (DPP-4) inhibitors are recommended for those at increased risk of hypoglycaemia, if not contraindicated (60, 62). GLP-1 receptor agonists are not recommended in those with an unexplained weight loss. The potential risks of worsening urinary incontinence, volume depletion, genital fungal and urinary tract infection with SGLT-2 inhibitors need to be discussed. The evidence of cardiovascular safety and potential benefit for older adults with DM is accumulating for GLP-1 receptor agonists and SGLT-2 inhibitors. Definitive data on cardiovascular safety for DPP-4 inhibitors use in older adults with DM is still pending (60). Long acting sulphonylureas and other insulin secretagogues should be avoided while short acting formulation, if considered, should be used cautiously (60, 73). Thiazolidinediones should be used very cautiously, if used at all, especially in those with heart failure, osteoporosis, falls or fractures, and macular oedema (60). Deintensification or simplification of oral antihyperglycaemic agents can be achieved by lowering or discontinuing some medications (60). Some patients may require insulin therapy when target glycaemic control are unmet with oral antihyperglycaemic agents. If so, aim to simplify and limit insulin use to a single morning basal dose and tailor according to individualised glycaemic target, level of functional dependency and supportive care availability. Long-acting basal insulin analogues are efficacious and safer in older people with DM compared to older basal insulin (60, 74, 75). Ironically, the likelihood of continued insulin is greater among frail patients (76), possibly reflecting the attitude and understanding of clinicians towards current pharmacological guidelines that are mainly oriented towards the management of non-frail patients. This is concerning as combined therapy with insulin and oral antihyperglycaemic agents is associated with significantly higher frailty levels (77). For those with type 1 DM, insulin therapy remains an essential life-preserving therapy. In older adults with DM, the recommended target blood pressure is <140/90mmHg in mild and moderate frailty and <150/90mmHg in severe frailty (60). Though few studies have examined differential outcomes in these patients. Further, DM and hypertension together in combination are associated with higher frailty prevalence (78). More evidence for lipid lowering therapy and aspirin use, especially for primary prevention, is also required.

Key challenges managing DM in frailty with suggestions and areas requiring additional research are presented in **Table 1**.

**Table 1:** Overview of key challenges and recommendations on the management of diabetes mellitus (DM) in frail older adults.

Aspects of Care	Challenges	Recommendations
***Screening for frailty***	Variable characteristics used to define frailty criteria Limited studies on psychometric properties and clinimetric performance of frailty screening tools in DM No instruments designed specifically for DM	Further research needed including diagnostic accuracy testing of new ‘bespoke’ instruments designed specifically to identify frailty in DM
***Assessment to confirm frailty and tailor interventions***	Cases are complex combining physical, cognitive, psychological, functional and social issues that should be assessed holistically (this approach is well established in geriatric medicine but less so in diabetes care)	Interdisciplinary approach using the principles of Comprehensive Geriatric Assessment (CGA) Need to research if CGA and ‘standard’ geriatric medicine interventions targeting frailty improve outcomes in DM or whether ‘bespoke’ CGA pathways are required for this population
***Glycaemic control***	Risk of hypo/hyperglycaemia in frail older adults Lack of data on how best to manage glycaemic control in different levels of frailty (a one size fits all approach may not be optimal)	Recommendation from 2021 Expert Consensus: Mild frailty: <58mmol/mol Moderate frailty: <64mmol/mol Severe frailty: <69mmol/mol Research is required to clarify if these targets improve outcomes in patients with frailty and DM across the spectrum of frailty
***Non-pharmacological intervention***	Choosing between dietary restrictions/weight loss versus adequate calorie/protein intake Individualising dietary plan and exercise intervention based on physical/cognitive status, personal preference and social/economic situation Concerns over effect of dietary modification in advanced frailty	Focus on optimal calorie and protein intake to prevent and treat frailty and sarcopenia Resistance exercises, balance training, community based/supervised fitness programs Research is required to clarify whether tailored nutritional plans can modify outcomes across the spectrum of frailty (prevent/reverse early frailty versus stabilize those with more advanced frailty understanding any potential trade off in quality of life)
***Pharmacological treatment***	Limited choice of oral antihyperglycaemic agents Poor understanding of treatment deintensification among some clinicians Difficulty adjusting and administering medication such as insulin Limited studies of pharmacological therapies for DM in those with advanced age or frailty	Metformin as first-line treatment, second-line options include GLP-1 receptor agonists, SGLT-2 inhibitors and DPP-4 inhibitors Avoid long acting sulphonylureas and other insulin secretagogues If insulin is necessary, use long-acting basal insulin analogues as a single morning dose Stop/reduce medications if suitable Opportunistically educate patients, caregivers and healthcare providers Additional research is required to examine the effects of pharmacological treatments in frail older patients with diabetes and whether benefits are sustained across the spectrum of frailty

## Supportive care

Given the often limited life expectancy of these patients, supportive care should be considered. Managing DM is difficult for frail older adults who may find adjusting insulin, choosing optimal times to take medications and meals, engaging with new technologies and recognizing signs of hypoglycaemia, challenging. Hence, treatment regimens should be simplified and caregivers encouraged to support management. Education for patients and caregivers is essential and may reduce care costs. Healthcare providers should opportunistically use each contact to educate and staff (including those of long term care and rehabilitation facilities) should likewise receive regular training on this complex topic (60, 62). Care should focus on symptoms and comfort in more advanced frailty and at end-of-life. Supported telemedicine and remote blood glucose monitoring may be considered if attending in-person reviews are difficult (79, 80). Blood glucose levels should be maintained at an acceptable range to prevent hypoglycaemia and acute complications of hyperglycaemia (60), ideally between 6-15 mmol/L (81). Advance planning involving patients and caregivers is recommended to acknowledge patient preferences and to avoid unnecessary or inappropriate interventions (68).

## Uncertainties and future directions of research

While research has advanced our understanding of the complex relationship between DM and frailty in older people, the exact underlying pathophysiology, bi-directional nature and optimal diagnostic and treatment strategies remain largely hypothetical and need further research. The trajectories and transitional states of frailty in older adults with DM are not established and need well-designed prospective studies and harmonised approaches to data collection. Most clinical trials of DM therapies primarily enrolled middle-aged participants and very few frail older adults (82). Similarly, few studies have compared the effectiveness and suitability of different classes of glucose-lowering agents in older patients (61). The benefits of other medication classes cannot easily be inferred from trials not specifically designed for this complex cohort of patients (59). The role of newer anti-hyperglycaemic agents, in particular those with less risk of hypoglycaemia or other adverse event among people with DM who are pre-frail and frail need further study, particularly in advanced frailty and end-of-life. In addition, there is limited evidence for how frailty can be prevented, reversed or managed in DM. This reflects the lack of data on optimal strategies to address frailty in general. Research is required to understand if there are consequences to managing both conditions in tandem such as the potential for over/under-treatment, drug-drug interactions and adoption of protocolized, one-size-fits-all management algorithms and whether these will be adhered to, if effective (83). There is also uncertainty over whether tailored, complex and multi-dimensional management strategies are acceptable to all patients.

Other research areas that require focus include examination of optimal frailty screening and diagnosis strategies, the relationship between frailty, DM and sarcopenia, how DM-related target organ damage evolves in frailty, how different clinical and HbA1c targets impact outcomes and translate into different costs, and the effects of new DM technologies on frailty (84).

In conclusion, frailty and DM are age-associated conditions that commonly co-exist. Frailty impacts the progression of DM, the intensiveness of its control and the selection of treatments. DM is associated with worse outcomes including increased mortality in frailty. Early detection of frailty in older people with DM provides the opportunity to consider targeted interventions to reduce disability and functional decline. A CGA should establish the objectives and optimal model of care. Although little fact-based knowledge on the effectiveness of existing and new treatments in frail diabetic patients is available, exercise and diet remain the cornerstone of the treatment of both entities. A detailed description of the evolution of each condition in the presence of the other is now warranted.

## Author contributions

All authors contributed to the article and approved the submitted version.

## Conflict of interest

The authors declare that the research was conducted in the absence of any commercial or financial relationships that could be construed as a potential conflict of interest.

## Publisher’s note

All claims expressed in this article are solely those of the authors and do not necessarily represent those of their affiliated organizations, or those of the publisher, the editors and the reviewers. Any product that may be evaluated in this article, or claim that may be made by its manufacturer, is not guaranteed or endorsed by the publisher.
